# Post-retrieval Distortions of Self-Referential Negative Memory: Valence Consistency Enhances Gist-Directed False, While Non-negative Interference Generates More Intrusive Updates

**DOI:** 10.3389/fpsyg.2021.668737

**Published:** 2021-06-25

**Authors:** Dong-Ni Pan, Xuebing Li

**Affiliations:** ^1^Key Laboratory of Mental Health, Institute of Psychology, Chinese Academy of Sciences, Beijing, China; ^2^Department of Psychology, University of Chinese Academy of Sciences, Beijing, China; ^3^Department of Cognitive Psychology, Institute of Cognitive Neuroscience, Faculty of Psychology, Ruhr University Bochum, Bochum, Germany

**Keywords:** reconsolidation, self-referential, episodic simulation, emotion, updating

## Abstract

According to the theory of reconsolidation, the contents of an original memory can be updated after reactivation with subsequent new learnings. However, there seems to be a lack of an appropriate behavioral paradigm to study the reconsolidation of explicit self-related memory, which is of great significance to further explore its cognitive neural mechanism in the future. In two separate experiments, we adapted a trial-by-trial interfering paradigm with a self-episodic simulation process and investigated (1) whether it is possible to reconsolidate negative memories under the new behavioral paradigm and (2) how the emotional valence of post-retrieval interference material affects the reconsolidation of negative memories. The results showed that the negative memories under trial-by-trial self-simulation can be degraded and updated *via* post-retrieval interference processes. Individuals whose original memories were reactivated by initial background cues and who were then presented with new interference situations were less able to recall original scenes and showed more memory intrusions on these scenes than those who had experienced new learning without reactivation or only reactivation without interference. Furthermore, the extent and manner of memory change/updating were greatly influenced by the characteristics of interference information. For memories with negative valences, new learning materials with the same valence produced superior interference effects in the form of lower correct recalls and more integrated false; whereas the neutral interference materials can cause more memory intrusion. Post-retrieval memory distortions of negative self-memory may underlie different functional mechanisms.

## Introduction

According to classical memory consolidation theory, information goes through a single consolidation process after being encoded, which converts short-term memory into long-term memory. Once this transformation occurs, these memories are considered permanent and are resistant to change (Mcgaugh, 1966). However, this traditional theory has recently been usurped by a new and more ecological theory, suggesting that memories are dynamic entities that can be destabilized by reactivation and can thus be vulnerable to further modifications (Nader et al., 2000). The role of reactivation in memory destabilization has been observed in different memory systems, such as fear conditioning (Nader et al., 2000; Schiller et al., 2010), procedural memory (De Beukelaar et al., 2014), and declarative memory (Hupbach et al., 2007; Chan and LaPaglia, 2013). Using various post-retrieval manipulations, namely, pharmacology (Kindt et al., 2009), behavior procedures (Hupbach et al., 2007; Schwabe and Wolf, 2009; Schiller et al., 2010), and neurostimulation (Censor et al., 2014), the original memory will experience post-retrieval impairment (Schwabe and Wolf, 2009; Schiller et al., 2010) or post-retrieval enhancement (St Jacques and Schacter, 2013; Censor et al., 2014). All these indicated that the initial memory can be substantially altered *via* a process of reconsolidation (see review Nader and Einarsson, 2010; Lee et al., 2017; Elsey et al., 2018). Indeed, several studies have revealed that the functional mechanisms of reconsolidation may stem from the process of updating existing memories with new information in order to maintain their relevance to our daily lives, thus reflecting a kind of organic adaptation (Schacter et al., 2011; Exton-McGuinness et al., 2015).

Negative memories usually have special significance for an individual, such as the adaptive function of warning to avoid danger, and were typically shown to be more resistant to be forgotten (Bowen et al., 2018); whereas maladaptive negative memories can also be the crux of many patients with affective disorder (Reynolds and Brewin, 1999; Foa et al., 2000). For example, individuals with posttraumatic stress disorder (PTSD) would experience unwanted memories through nightmares, flashbacks, or intrusive recollections of a traumatic event (American Psychiatric Association, 2013). Other mental illnesses, such as depression (Newby and Moulds, 2010) and phobia (Coles and Heimberg, 2002), can also be seen to have maladaptive emotional memories as a core feature. The framework of memory reconsolidation opens the door to the possibility that negative maladaptive memories might be modified in clinical practice (Schwabe et al., 2014; Phelps and Hofmann, 2019).

However, even if general *neutral* memory seems to be able to achieve reconsolidation through a variety of paradigms (Hupbach et al., 2007, 2009; Sandrini et al., 2013; Sinclair and Barense, 2018; Zhu et al., 2019), in existing reconsolidation studies, there are inconsistent results on whether *negative* declarative memories can be updated. For instance, Schwabe and Wolf (2009) found that episodic learning of new unrelated content (a complex story with relatively neutral content) could interfere, *via* reconsolidation processes, with neutral autobiographical memories but not with negative ones. Pineyro et al. (2018) found that, only in women samples, negative autobiographical memories can be modified through the experience of positive information after a reactivation procedure. Kredlow and Otto (2015) only revealed a very small effect of post-retrieval intrusion for real negative memories relating to bombings, especially when interference materials were neutral or positive. Meanwhile, Wang et al. (2021) brought some positive results that aversive episodic memory can be significantly impaired *via* reactivation-updating procedures, even with some unwanted side effects. These results suggest that the reconsolidation of negative memory can occur but that the occurrence would indeed be subject to certain restrictions; so, it is important to find out under what conditions negative memory reconsolidation is more likely to be induced, which actually can be the key to leading memory editing into real application practice.

We noted that traditional tasks that explore naturalistic (autobiographical) negative memory typically work by collecting personal memory prior to the experiment and using this as the evaluation material. Such a method, however, by not allowing for sufficiently precise control of the nature of memory materials (e.g., age, rehearsal level), seems to limit the in-depth examination of the current issue; On the other hand, laboratory designs on episodic negative memory reconsolidation usually contained items that are essentially context-independent as learning materials (isolated objects, pictures, or videos; e.g., Chan and LaPaglia, 2013; Wirkner et al., 2015). More importantly, the memory/retrieval process lacked the involvement of explicit autonoetic awareness, leading to the difficulties in characterizing the unique self-processing of episodic memory reconsolidation. Indeed, psychological philosophers have long associated the self with memory, believing that the formation of self-awareness depends on individual memory and that the dynamic construction of memory depends on self-processing (Conway and Pleydell-Pearce, 2000; Conway et al., 2004). In the self-memory system (Conway and Pleydell-Pearce, 2000; Conway, 2005), objective information is thought to be selectively encoded, consolidated, and reconsolidated to form personal records, which is substantially directed by self-representation. Thus, there seems to be a lack of a design with both high material standardization and high self-involvement to realize the investigation of self-memory reconsolidation.

Interestingly, the process of remembering past experiences is closely related to, or even overlaps with, the process of imagining possible future events. Episodic memory and episodic future simulation represent two aspects of mental time travel (Suddendorf and Corballis, 1997) and share similar neural mechanisms (Wheeler et al., 1997; Preston and Wagner, 2007). Moreover, there is a precedent for using future simulations to simulate memory construction (Szpunar et al., 2012). Through imagining procedures, it is possible to produce standardized, but self-related, memory materials. This ensures a relatively homogeneous autobiographical content, which is a crucial aspect for gaining a deeper insight into the phenomenon of episodic memory reconsolidation. In addition, to highlight the context (spatial–temporal) dependence of memory, the paired-associate learning paradigm can be referenced (Larrabee, 2000). In this paradigm, A–B combination is used as learning material, A–D learning is used as an intervention, and C–D learning is used as control manipulation, in which A plays a role in memory reactivation reminder. This way, each event memory (i.e., B associated with A) should be triggered by a specific context cue (i.e., A) rather than getting the semantic knowledge of an item memory (i.e., general B). In addition, under this trial-by-trial design, the reactivation process of each event (e.g., A1-, A2-…) and the post-retrieval intervention process of each event (e.g., -D1, -D2,…) could also be independently examined.

To summarize, in this study, we would develop a new paradigm using self-referenced episodic simulation stages as encoding/interfering phases and dismountable visual scene combinations as memory materials, with the aim of resolving two issues regarding memory reconsolidation. First, can negative declarative memory be reconsolidated under self-reference processing? Second, what kind of interference information is required to more effectively update negative memory? For the second question, we devoted a particular focus to the valence of interference information or the consistency in valence between the original memory and the interference information. This is because previous studies seemed to show some complex and interesting inconsistencies on this issue. For example, some suggested that memory reconsolidation is only possible if the interfering material has similar valence characteristic to the original memory material, such as studies by Schwabe and Wolf (2009) demonstrating that neutral information can interfere with neutral memory only (but not for negative ones) and further studies by Kredlow and Otto (2015) showing that only stories with negative content could interfere with negative memories. Other recent data showed that positive or neutral information can also interfere with original negative memories possibly *via* different routes (Pineyro et al., 2018; Wang et al., 2021). In fact, investigating the valence characteristics of the interfering materials after reactivation has an important guiding significance for intervention strategies in real-world applications. Even though predecessors mentioned above have made exploratory attempts on this issue, more studies still need to be processed to clarify how the interaction pattern of original memory valence and interfering material valence affects the final outcomes of memory retention.

Specifically, we carried out two experiments. In Experiment 1, we examined the possibility of negative self-memory updating through reconsolidation. In this experiment, the participants learned a certain number of negative pictures through self-referential simulation on the first day, with all pictures divided into two parts: the “cue object,” acting as a background/contextual clue for a certain scene, and the “core object,” acting as the remaining content to form the complete scene. Then, the participants were randomly divided into three manipulative groups after 48 h: (a) Reactivation-Interference, Re-I; (b) no Reactivation-Interference, noRe-I; and (c) Reactivation-no Interference, Re-noI. We hypothesized that the Re-I group, where the original episodic memory is reactivated by presenting the initial “cue object” and, at the same time, input with interference information (also with self-referential style), would have the worst memory performance for original learning. In Experiment 2, we examined the ways to optimize the phenomenon of reconsolidation, specifically, how the valence combination of initial and interference materials affects the degree of updating. In this experiment, the within-subject design was mainly employed. The participants were all conducted with Re-I manipulation (i.e., all of the participants were reactivated and interfered), but the learning (initial memory) materials and interference materials included different valence combinations (i.e., Neutral learning–Neutral interference; Neutral learning–Negative interference; Negative learning–Negative interference; Negative learning–Neutral interference). According to previous dominated conclusions on autobiographical memory (e.g., Schwabe and Wolf, 2009; Kredlow and Otto, 2015), we hypothesized that the consistent valence between the original memory and the interfering information would lead to the greatest number of memory errors or the memory updating. At the same time, considering the recent data (Pineyro et al., 2018; Wang et al., 2021), we also held an open attitude toward the interference effect of neutral materials on the current self-simulation paradigms.

## Experiment 1

### Aim

In this experiment, we first aimed to verify whether negative declarative memories under self-reference simulation can be updated *via* a post-retrieval interference procedure.

### Methods

#### Participants

All the participants were recruited through an online participant recruitment platform and were required to complete a basic screening questionnaire (including demographic information and medical history). Individuals who reported being diagnosed with a mental illness (schizophrenia, major depression, bipolar disorder, anxiety-related disorders) were excluded from the sample. The sample size was evaluated through power analysis using G*Power 3.1. Based on previous studies that refer to post-retrieval updating of episodic memory (e.g., Chan and LaPaglia, 2013; Kredlow and Otto, 2015), in which the mean effect size on reconsolidation associated amnesia/distortion *d* ~ 0.55, calculations indicated that at least 53 participants in each group would be required to achieve 80% power. Since this experiment was conducted on an online platform, some data may not pass quality control, so the number of participants was increased appropriately during the recruitment period. Two hundred and fifty participants (aged 18–30) took part in the online experiments, and 214 completed the whole experimental process according to the experimental requirements and passed data quality control. They were randomly assigned into three groups based on the computer random seeds (Re-I: reactivation-interference, *n* = 76; Re-noI: reactivation-no interference, *n* = 62; noRe-I: no reactivation-interference, *n* = 76). The groups of the participants showed no significant differences in age [for years, Re-I: 22 ± 2.88, noRe-I: 22.7 ± 2.85, Re-noI: 22.35 ± 3.16, *F*_(2)_ = 1.09, *p* = 0.342], gender ratio (for male rate, Re-I: 35.93%, noRe-I: 50.81%, Re-noI: 45.76%, *χ*^2^ = 3.43, *p* = 0.179) or education level [for years, Re-I: 14.96 ± 2.64, noRe-I: 15.62 ± 2.74, Re-noI: 15.22 ± 2.78, *F*_(2)_ = 1.17, *p* = 0.324]. All the participants provided informed consent through the electronic form before the experiment and received a small payment as compensation. The research was approved by the ethics committee of the Institute of Psychology at the Chinese Academy of Sciences and was carried out in accordance with the approved guidelines.

#### Task Procedure

**Figure 1:**
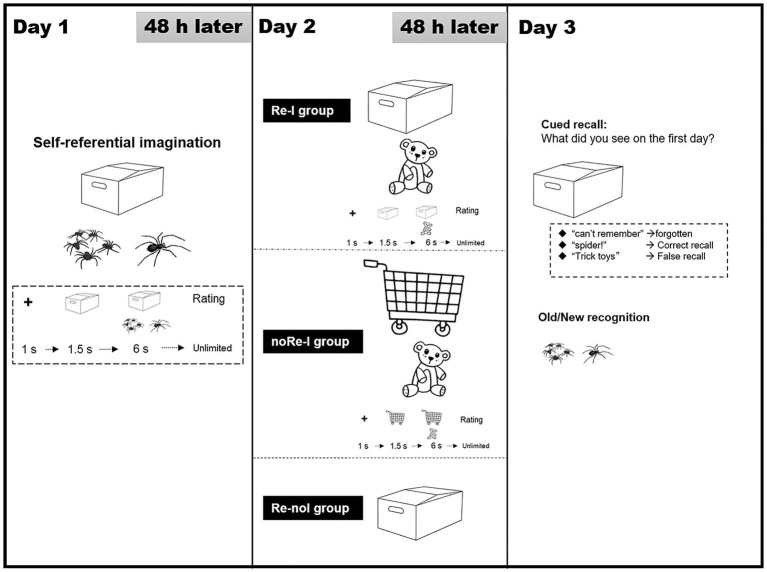
Program flow diagram of Experiment 1. The experiments consisted of three experimental sessions (learning, interfering, and testing) with 48-h intervals between each. On Day 1, all the participants learned a series of negative scenarios (each one consists of the cue object/background and the core object) trial by trial through self-referential simulation, and rated self-involvement level. On Day 2, the participants were divided into three groups randomly: (a) Reactivation-Interference group, Re-I, in which the first-day memory was reactivated on the participants *via* the presenting the same cues as Day 1 and it interfered with the rematch scenarios with old cues and novel objects; (b) Reactivation-no Interference group, Re-noI, in which the first-day memory was reactivated on the participants *via* presenting the same cues as Day 1, but no novel information were additionally presented; and (c) no Reactivation-Interference group, noRe-I, in which the old memory was not reactivated in the participants deliberately but they were presented novel backgrounds and core objects, even though these novel objects were the same as that in the Re-I group. The participants in the Re-I and no Re-I groups were required to learn these scenarios by a self-simulation manner and report self-involvement. On test day, all the participants underwent cued recall test and recognition test for the first day of learning. In the cued recall test, the cue object/background of the first day were presented to require the recall of the paired objects of the participants in Day 1. The answers of the participants were divided into three categories: (1) forgotten, (2) correct recall, and (3) false recall.

The formal task consisted of 3 experimental days (learning, interfering, and testing) with a 48-h interval between each experimental day (see Figure 1 for experiment diagram).

##### Day 1: Learning *via* Self-Referential Simulation

All the participants were required to view and visualize 44 negative scenes using a self-referential method. Each scene included two separate components: (a) the cue object and (b) the core object. The cue object of the scene provided context/background information of the event and had no emotional valence (e.g., a box of a specific shape), while the core object illustrated the key content of the event and had a negative valence (e.g., spiders). Together, the two components (the cue object plus the subsequent core object) constituted a scene with a specific meaning, which was designed to induce an unpleasant mood in the participants. All scenes were selected from a pool of pre-prepared candidate materials (120 scenes), which had been pre-rated by 50 independent college students (21 females, age = 22.1 ± 4.9) according to their degree of discernibility (“how well did you recognize the images in the scene”), familiarity (“how often have you experienced this scene”), and valence (“how much pleasure was experienced while viewing the scene”), and the relevance between the core object and the cue object of a scene (“to what extent can the cue background and the later object be connected in mind”). All measures were seven-point scales. Scenes with more than six points of familiarity (*n* = 12) and less than two points of discernibility (*n* = 12) were excluded. In addition, for each of the selected 44 materials used on Day 1, the mean rating (the average score from all raters) of the valence rating (one for very unhappy, seven for very happy) should fell between one and three points, such that the average valence level of the 44 scenes was 2.19 ± 0.76 (*n* = 52 was excluded). In the end, the mean familiarity of the selected materials was 3.47 ± 0.45, and the mean discernibility was 5.34 ± 0.78. The relevance of the core objects within the cue objects remained at a medium level (mean = 4.18 ± 0.78).

The participants, on the first learning day, were given the specific instruction: “Now, we will present some scenes, each consisting of two parts: the cue object serving as background information will appear first, and then the core object will be presented. After the core object is rendered, the whole scene will remain on screen for 6 s. During this time, you need to imagine yourself interacting with the core object in the scene. Since these scenes are simple stick figures, you will have plenty of mental room for visualization, but remember to do the self-referential imagination within the basic depiction of the scene.” Each participant was given two exercise trials comprising materials that were different from the formal experiment. The process of trial-by-trial viewing and simulating was repeated two times. During the second simulating round, the participants were asked to rate the level of self-involvement/visualization that they experienced at the end of each trial on a seven-point scale (the rating instruction was “How much do you involve yourself in the process of imagining the scene so that the simulation becomes immersive”). For each trial, the procedural sequence was as follows: first, a fixation point was presented for 1 s, then a cue object was presented for 1.5 s, followed by a core object, which was presented for 6 s; after which the participants were required to provide their involvement/visualization rating on the second simulating round.

##### Day 2: Reactivation and Interference

Approximately 48 h (49.6 ± 3.26) after the initial learning day, all the participants were randomly assigned to one of the three groups that differed according to how cue objects and core objects were combined.

###### Reactivation-Interference Group

For the Re-I (reactivation-interference) group, we presented the participants with 44 interfering scenes. Specifically, although the cue objects were the same as the original scenes seen on Day 1 (e.g., a box of a specific shape), the core objects were altered to objects that were novel and neutral (e.g., a toy bear). Indeed, the 44 original cue objects and the 44 novel core objects formed 44 independent scenes with non-negative valence. All scenes were pre-rated (along with other 60 scenes) according to their degree of discernibility, familiarity, valence and the relevance between the cue object and core object (seven points) by another 50 independent college students (20 females, age = 21.1 ± 7.2). The mean valence rating for each scene fell between three and six points, and the average valence level of the 44 scenes was 4.95 ± 0.86. In addition, the cue object–core object relevance of these materials was maintained at a moderate level (grand mean = 5.17 ± 0.38, slightly higher than the negative scenes on Day 1, because negative scenarios are less likely to occur in real life). None of the scenes in the material were less than two points of discernibility or with more than six points of familiarity, with a mean familiarity of 4.17 ± 0.45 and a mean discernibility of 5.32 ± 0.88.

Similar to the learning scenario from Day 1, participants were asked to view and visualize the scenes self referentially for two repeated runs. The procedure took the following sequence: a fixation point was presented for 1 s, then the original cue object was presented for 1.5 s, and finally, the core object was presented for 6 s. Again, the involvement/visualization rating only occurred during the second run. We presumed that, by presenting the original cue objects first, the participants would reactivate the original learned scene. In this way, the introduction of new information (novel core objects) following the old cue object would create a prediction error, thus exerting an interference effect on the original memory trace and rendering it unstable.

###### No Reactivation-Interference Group

For the noRe-I (no reactivation-interference) group, we presented the participants with 44 novel interfering scenes; but for this group, we did not deliberately reactivate any memory trace from the scenarios seen on Day 1. Specifically, for this group, we used novel materials for cue objects (e.g., a shopping cart), despite core objects being new and identical to those used for the Re-I group (e.g., a toy bear). Similarly, these novel control scenes were also rated on their degree of discernibility, familiarity, valence, and relevance of two parts (seven points) by another 30 independent college students (10 females, age = 21.1 ± 7.2). The mean valence rating of each scene also fell between three and six points, and the average valence level of the 44 scenes was 4.89 ± 0.87. The cue object–core object relevance of these materials was maintained at a moderate level [grand mean = 5.07 ± 0.4, with no difference to corresponding interference materials, *t*_(43)_ = 0.68, *p* = 0.511]. In addition, none of the scenes in the material were with low discernibility or overly familiar, with a mean familiarity of 4.21 ± 0.51 and a mean discernibility of 5.32 ± 0.61. The participants were asked to view, visualize, and rate in a self-referential manner using the same procedural design as was applied to the Re-I group.

###### Reactivation-No Interference Group

For the Re-noI (reactivation-no interference) group, the participants were required to perform a simple memory reactivation process without new learning. Specifically, we presented these participants with 44 scenes in which the original cue objects from Day 1 were visible, but the corresponding original core objects were concealed. The trial sequence was as follows: fixation point (1 s), original cue object (1.5 s), and scene with the concealed core object (6 s). The participants were asked to simply pay attention to the scenes and were not given any instruction regarding self-referential imagination.

##### Day 3: Unexpected Memory Tests

Approximately 48 h later (50.1 ± 3.98), all the participants were given an unannounced test examining their memory of scenes from Day 1. First, the participants performed a cued recall test. Specifically, subjects were presented with the cue objects of scenes presented on the first day, and the participants were asked to report the core objects that were associated with them. The participants were told that it was important to only remember Day 1 items and that no self-referential requirements were needed. They did this by typing their response onto a keyboard without any time limit and free of self-imagination. Next, the participants performed a recognition test in which all of the core objects from Day 1 were presented and randomly mixed with new objects of similar proportions (*n* = 44). The new core object items were selected from the same categories as the learning materials from Day 1 (e.g., weapons, animals, wounds, etc.). Independent ratings (conducted by same raters as learning materials) for pleasure ratings fell between one and three points with an average rating of 2.13 ± 0.77. The participants were asked to judge whether the core objects had been presented before or whether they were new, with no self-referential viewing required.

#### Experimental Platform and Data Quality Control

The experimental program was written by the GODOT game engine^1^ and exported as executable files that could run on multiple terminals (including Windows, IOS, and Linux). All the participants were required to install the program successfully on their computers. The participants were told that they could open the program and perform the task at their convenience, but they had to make sure that they had the accessibility to complete the 3-day sequence with an interval of 48 h (±2 h). Before the experiment, the experimenter explained and emphasized the experimental operations for each participant (e.g., ensuring a quiet task environment, conducting the experiment according to the prescribed time, following the requirements of the experimental instructions). During the experiments, guided instructions and exercises were carefully set up in the program to enable the participants to understand and perform prescribed operations. The grouping of the participants was randomly determined by the computer. All generated data, including ratings, recordings, and test results, were stored in a user-specified folder and encrypted. The participants were required to send the result file to the experimenter once they completed the whole experiment. If a participant failed to complete the 3-day task, no result file will be generated. Before the experiment was completed, the participants did not know the purpose of the experiment. They were told that it was a task to imagine and rate the picture material. The memory test was unexpected. In addition, the experimenter coding the answers did not know the group information of the participants until the final statistics.

Since the whole experiment process was not strictly supervised by the experimenter, quality control was additionally conducted on the acquired data to eliminate the influence brought by “inattentive participant.” Specifically, participants (*n* = 250) meeting the following criteria were excluded for analysis: (1) whose ratings, recordings, and test data were incomplete (*n* = 3); (2) who watched the instructions for less than 3 s (*n* = 4); (3) whose average rating RT was less than 1 s and the rating score was a fixed value (*n* = 14); and (4) whose hit rate of recall test was less than 25% and whose forgetting rate was higher than 60% (*n* = 15).

#### Data Analysis

First, we performed a one-way ANOVA to test whether there was any initial difference in self-involvement/visualization rating during simulating/encoding among the three groups on Day 1.

For the recall test data collected on Day 3, all responses pertaining to scenes viewed from Day 1 were divided into three categories: (1) correct recall, recall the correct core object matching its corresponding cue; (2) false recall, report an incorrect core object; and (3) forgotten recall, explicitly declare not remembering. The classification of responses (1, correct recall; 2, false recall; 0, forgotten) was performed independently by two experimenters with a high rate of consistency [Cramer’s V_(6072)_ = 0.99, *p* < 0.001]. Inconsistent judgments (<20) were identified, re-assessed, and confirmed. Ratio parameters (number of correct/forget/false recall items divided by the total number, i.e., 44) were calculated. For recognition tests, we calculated the general accuracy.

We conducted one-way ANOVA and *post-hoc* analyses corrected by the Bonferroni method and a *t*-test (two tails) to reveal alterations in memory performance among the three groups. The descriptive statistics presented are in the form of mean ± SD. To explore the relationship between memory retention and self-involvement during coding and interfering, we performed Pearson’s correlation analyses among memory indicators and self-involvement rating (on Day 1 and Day 2).

### Results

We observed no differences between the groups in self-involvement/visualization on Day 1 [Re-I: 3.87 ± 1.13, noRe-I: 4.11 ± 0.91, Re-noI: 3.98 ± 0.87, *F*_(2)_ = 0.904, *p* = 0.407], suggesting no encoding differences among the three groups at the initial learning stage.

**Figure 2:**
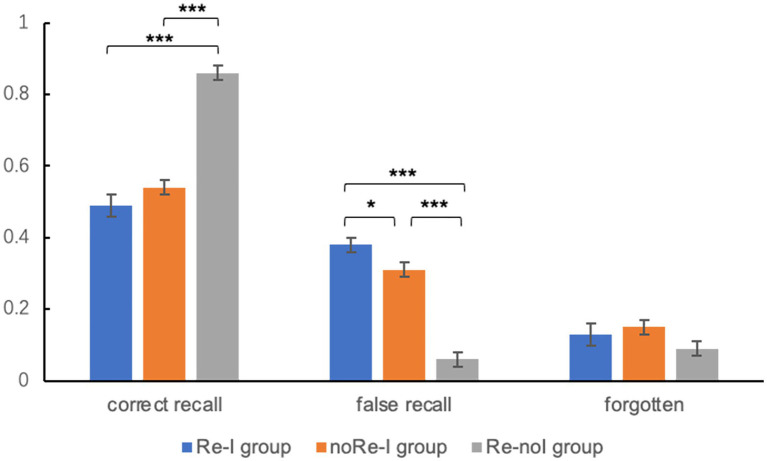
Memory performance of cued recall tests among three groups (Experiment 1). For acronym, noRe-I represents the no Reactivation-Interference group; Re-noI represents the Reactivation-no Interference group; and Re-I represents the Reactivation-Interference group. For the results, the correct recall for the Re-noI group was significantly higher than that of the noRe-I and Re-I groups. The false recall for the Re-I group was significantly higher than that of both the Re-noI and noRe-I groups. The error bar in the figure represents the standard error. ^*^represents *p* < 0.05 and ^***^represents *p* < 0.001.

For cued recall test on Day 3, we found significant differences in memory performance between the three groups [for correct recall, *F*_(2)_ = 40.92, *p* < 0.001; false recall, *F*_(2)_ = 37.23, *p* < 0.001]. *Post-hoc* analyses showed that correct recall for the Re-noI group (0.85 ± 0.17) was significantly higher than that produced by both the Re-I group (0.49 ± 0.25, *p* < 0.001) and the noRe-I group (0.54 ± 0.27, *p* < 0.001). The correct recall for the Re-I group was lower than that produced by the noRe-I group but was not statistically significant (*p* = 0.166). Nevertheless, the false recall for the Re-I group (0.38 ± 0.22) was significantly higher than that of both the Re-noI (0.06 ± 0.1, *p* < 0.001) and noRe-I groups (0.31 ± 0.27, *p* = 0.047). For the forgetting rates, no significant main effect was revealed [*F*_(2)_ = 1.3, *p* = 0.13, Re-I: 0.13 ± 0.17, noRe-I: 0.15 ± 0.15, Re-noI: 0.09 ± 0.17]. The results recall performances among the three groups were visualized in Figure 2. For the recognition test, however, no significant main effect of group was found [*F*_(2)_ = 2.09, *p* = 0.199, Re-I: 0.87 ± 0.13, noRe-I: 0.89 ± 0.1, Re-noI: 0.9 ± 0.08].

Pearson’s correlation analyses revealed that, only for the Re-I group, the self-involvement rating on Day 2 positively correlated with the false recall [*r*_(76)_ = 0.27, *p* = 0.018], indicating that self-involvement during post-retrieval interfering can profoundly affect the retention of the original memory.

### Discussion

In this section, we have shown that episodic memory can be reconsolidated and updated in the self-referential simulation paradigm. This is reflected by the finding that the false recall of the Re-I group (in which memory was reactivated followed by a new learning interference) was significantly higher than that of both the Re-noI (in which memory was reactivated without new learning) and noRe-I groups (in which no memory reactivation was conducted). These results indicated that the *reactivation* of initial memory and the input of new information (*interfering*) are both crucial to updating a memory trace, not only in typical declarative learning (Hupbach et al., 2007; Chan and LaPaglia, 2013; Rodriguez-Ortiz and Bermudez-Rattoni, 2017) but also in the current situation, where self-simulation was the way to learn. Besides, the positive correlation between self-involvement rating on interference day (Day 2) and rates of false recall provides further confirmation of this phenomenon and suggests the importance of post-retrieval self-processing in memory updating.

The experimental manipulation that was specific to the Re-noI group relates to memory reactivation without new information input. This produced improved memory retention compared to the Re-I and noRe-I groups. The memory advantage demonstrated here is, to some extent, consistent with a classic retrieval practice effect (Karpicke and Roediger., 2008). The familiar background pictures implicitly trigger the participants to extract and practice the original memory, which incidentally promotes the retention of its content, thus producing higher hit rates. This post-retrieval memory enhancement indeed can also imply some reconsolidation mechanisms in line with Forcato’s series studies (Forcato et al., 2011, 2013, 2014, 2016), where memory persistence improvement was stably observed as outcomes of reminder presentation. All these indicated the important role of reminders to destabilize memory traces.

Caution is advised in drawing the conclusion on whether the memory performance of the noRe-I group or Re-I group was better. To be specific, although the Re-I group showed more false recall than the noRe-I group (*p* < 0.05), the difference in the correct recall items of these two groups did not reach a significant level, given that the noRe-I group had relatively higher forgotten reports. Indeed, the retrieval process in the Re-I group, despite subsequent interference (leading more memory false), may still lead to a certain degree of enhancement of the original memory, suggesting the bidirectional effect of retrieval on memory outcomes (Forcato et al., 2013; St Jacques and Schacter, 2013). The absence of explicit reactivation of the initial memory, which was implemented in the noRe-I group, might serve as a typical strategy for natural negative memory fading (more forgotten items). Indeed, pure interference effect without reactivation (e.g., retroactive inhibition) can also be an important mechanism of memory impairment (Earhard, 1976). On the other hand, even without explicit reactivation manipulation, the noRe-I group may still implicitly extract the original memory trace because of the similar learning mode or experience some spontaneous reactivation. Therefore, the difference in memory retention between the Re-I and noRe-I groups requires more repeated verification.

In this experiment, the characteristic of learning/interference material was not emphasized. We observed that reactivation of initial memory plus neutral interference facilitated the updating of negative memory compared with the other two manipulations, but, in order to optimize the negative memories updating *via* post-retrieval processes, it is of great importance to examine the interference features of new learning (e.g., valence consistency mentioned in introduction) on the effect of memory reconsolidation, which can be conducted in Experiment 2.

## Experiment 2

### Aim

In this experiment, we would like to repeat the results of Experiment 1 in another independent sample. Given the clear beneficial memory effects demonstrated by the Re-noI group in Experiment 1, in the current experiment, only the Re-I and noRe-I groups were included. More importantly, we aimed to investigate whether the valence consistency between initial learning and post-retrieval interference has an effect on memory updating. Therefore, in group Re-I, the within-subject design was specially adopted, and neutral and negative materials were included in both the original memory learning phase and the interference phase, respectively, to form four valence combinations: (1) Neutral interference with Neutral origin (NeuO-NeuI); (2) Neutral interference with Negative origin (NegO-NeuI); (3) Negative interference with Neutral origin (NeuO-NegI); and (4) Negative interference with Negative origin (NegO-NegI). We determined that such a design could reveal an interaction between original memory valence and interference valence in a more effective and sensitive manner. It should be noted that, like Experiment 1, all learning phases were done through self-episodic simulation to enhance the self-referential nature of memory.

### Methods

#### Participants

We screened the participants in the same way as in Experiment 1. Eighty-eight college students participated in the experiment. Seventy-nine subjects completed the whole process and passed the data quality control. They were randomly assigned into the reactivation-interference group (Re-I group, *n* = 35) and the no reactivation-interference group (noRe-I, *n* = 44) by computerized draw. There were no significant differences in age [for years, Re-I: 22.77 ± 3.09, noRe-I: 22.29 ± 2.9, *t*_(77)_ = −0.79, *p* = 0.434], gender ratio (for male rate, Re-I: 48.57%, noRe-I: 40.91%, *χ*^2^ = 0.87, *p* = 0.774), or education level [for years, Re-I: 14.68 ± 2.55, noRe-I: 14.61 ± 2.69, *t*_(58)_ = 0.12, *p* = 0.351] between groups. All the participants checked the electronic informed consent form before the experiment and received a small payment as compensation.

#### Task Procedure

**Figure 3:**
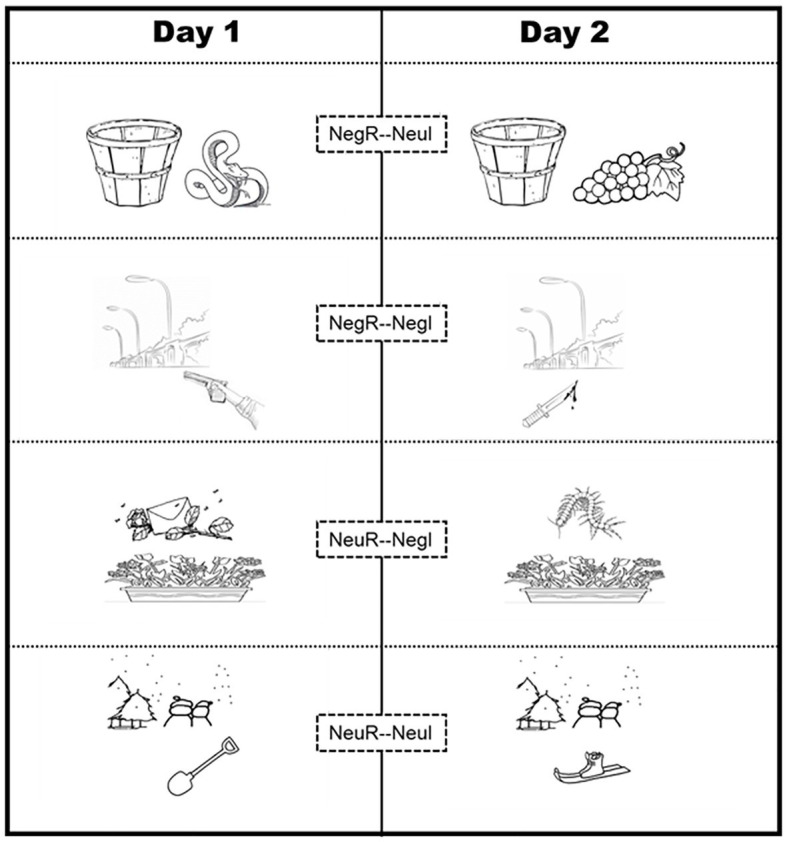
Example materials under four conditions in Experiment 2. All learning (Day 1)/interfering (Day 2) materials consisted of the cue object and the core object (which actually determine the core valence of the scenario). For all background cues from Day 1 neutral scenarios, half was matched with Day 2 neutral interference objects to form novel neutral scenarios and half was matched with Day 2 negative interference objects to form novel negative scenarios. The same matching rule applied 32 background cues from Day 1 negative scenes. Thus, all scenarios (on Day 1) were actually divided into four categories: (a) Negative origin with Neutral interference (NegO-NeuI); (b) Negative origin with Negative interference (NegO-NegI), (c) Neutral origin with Neutral interference (NeuO-NeuI), and (d) Neutral origin with Neutral interference (NeuO-NeuI). Within-subject design, thus, can be used to more sensitively investigate the effects of original memory valence and interference valence on memory reconsolidation.

The task procedure was similar to that in Experiment 1 and consisted of three experimental sessions (learning, interfering, and testing) with 48-h intervals between each. However, in contrast with Experiment 1, we additionally adopted a within-subject design for the Re-I group, whereby the scene valence of learning materials was manipulated on Days 1 and 2 to form 2 × 2 valence conditions, (1) Neutral interference with Neutral origin (NeuO-NeuI, *n* = 16); (2) Neutral interference with Negative origin (NegO-NeuI, *n* = 16); (3) Negative interference with Neutral origin (NeuO-NegI, *n* = 16); and (4) Negative interference with Negative origin (NegO-NegI, *n* = 16; see Figure 3). In addition, in order to compare the post-retrieval updating effect, we included the noRe-I group as a control group.

##### Day 1: Learning *via* Self-Referential Simulation

All the participants were required to view and visualize 64 scenes using the same self-simulation method as previously outlined. As with Experiment 1, each scene consisted of two separate components: (a) the cue object and (b) the core object. Unlike Experiment 1, these scenes comprised 32 neutral (neutral cue object + neutral core object) and 32 negative (neutral cue object + negative core object) scenes. These were selected based on pre-rated levels of discernibility, familiarity, valence, and object relevance. For each of the neutral scenes, the mean scores (the average score from all raters) for valence ratings fell between three and six points, and the average valence of the 32 scenes was 4.27 ± 0.99. For the negative scenes, the mean scores fell between one and three, with an average valence of 2.02 ± 0.88 (rated by the same group from Experiment 1). Participants were also asked to report their own level of self-involvement/visualization on a seven-point scale.

##### Day 2: Reactivation and Interference

Approximately 48 h (47 ± 2.15) after the initial day of learning, the participants underwent reactivation-interference manipulation or simply interference learning without deliberate reactivation.

###### Reactivation-Interference Group

Participants in the Re-I group were presented with cue objects from Day 1, which were intended to reactivate their original memory of the scene. However, in order to interfere with reactivations, each scene was then presented with a new core object. The participants were asked to view and visualize the scenes in a self-referential way and then provide a self-involvement rating.

Interference materials for Day 2 not only included 64 scenes (32 neutral, average valence rating = 4.11 ± 0.79; 32 negative, average valence rating = 2.14 ± 0.78) with cue objects identical to those from Day 1 but also included 64 novel core objects to be combined with cues to form the new scenes. For the 32 cue objects from Day 1 neutral scenes, one half of them were matched with Day 2 neutral interference core objects to form neutral scenes and the other half of them were matched with Day 2 negative interference core objects to form negative scenes. The same matching rule applied 32 cue objects from Day 1 negative scenes. Thus, for the participants in the Re-I group, Day 2 scenes were actually divided into four categories: (1) Neutral interference with Neutral origin (NeuO-NeuI, *n* = 16); (2) Neutral interference with Negative origin (NegO-NeuI, *n* = 16); (3) Negative interference with Neutral origin (NeuO-NegI, *n* = 16); and (4) Negative interference with Negative origin (NegO-NegI, *n* = 16). There were no significant differences in the discernibility and familiarity ratings between the four categories (*F*s < 1.5, *p* > 0.1).

###### No Reactivation-Interference Group

For the noRe-I (control) group, the participants were presented with 64 novel scenes that did not contain materials that could deliberately reactivate memories from Day 1. Both cue objects and core objects were novel for the individuals in this group, even though the core objects were the same as those used in the Re-I group. Again, the participants were also asked to view and visualize each scene in a self-referential manner and then provide a self-involvement rating.

The control materials for Day 2 contained 64 new cues to be combined with 64 core objects of the interference materials from Day 2 (including neutral and negative core objects) to form another 64 separate scenes (32 neutral, average valence rating = 4.1 ± 0.74; 32 negative, average valence rating = 2.12 ± 0.7). The noRe-I control group had to learn these materials on Day 2.

##### Day 3: Unexpected Memory Tests

Approximately 48 h (48 ± 1.15) after the second session, all the participants were given an unannounced test examining their memory of scenes from Day 1. The procedure is the same as that carried out in Experiment 1.

Testing materials consisted primarily of 64 cue objects from Day 1 (as a recall cue for the free recall test) and another 32 novel core objects (16 neutral and 16 negative, for the old/new recognition test). The new core object items were selected from the same categories as the Day 1 learning materials.

#### Experimental Platform and Data Quality Control

Similar to Experiment 1, the experimental program was written by the GODOT game engine^2^ and exported as executable files that could run on multiple terminals. All the participants were required to install the program successfully on their computers. The online guidance and data exclusion criteria of the participants were consistent with Experiment 1. Of all the participants, two were excluded for the too-short RT for rating responses, and seven were excluded for too many forgetting reports.

#### Data Analysis

In order to examine the self-processing differs between groups and conditions, three-way ANOVAs: 2 (Day: learning day, interfering day) × 2 (valence: neutral, negative) × 2 (group: Re-I, noRe-I) were performed for the self-rating data.

The memory performance indicators of recall performance included correct recall rate, false recall rate, and forgotten rate as Experiment 1. Notably, in this experiment, all false recall items were further divided into two types: (a) integrative false recall (integF) provides integrative errors to the given cue objects (e.g., a box + spiders on Day 1, a box + teddy bear on Day 2, an integrative error could be reporting a trickery toy) and (b) intrusive false recall (intruF) provides specific day-2 items as the original ones (e.g., a box + spiders on Day 1, box + teddy bear on Day 2, an intrusive error could be reporting a toy bear). The classification of responses (1, correct recall; 2, integrative false recall; 3, intrusive false recall; 0, forgotten) was performed independently by two experimenters with a high rate of consistency (Cramer’s V = 0.89, *p* < 0.001). Inconsistent judgments were identified, re-assessed, and confirmed.

In the current within-subject design, there were four scene conditions within the Re-I group according to the material types (NeuO-NeuI, NegO-NeuI, NeuO-NegI, and NegO-NegI), so, we performed a 2 (origin valence: neutral/negative) × 2 (interference valence: neutral/negative) repeated measure ANOVA for these memory indicators to reveal the impact of memory/interference valence on memory updating.

To replicate the results of Experiment 1, we performed an independent sample *t*-test (two tailed) between the Re-I group and the noRe-I group to test whether post-retrieval interference caused more memory false than the interference without memory reactivation.

### Results

For self-involvement ratings, the main effect of valence was significant, *F*_(1,77)_ = 42.16, *p* < 0.001. Self-involvement ratings on neutral scenes were significantly higher than those on negative scenes. The interaction between valence and day was significant, *F*_(1,77)_ = 16.96, *p* < 0.001. The simple effect analysis showed that the rating difference on valence (neutral-negative) was smaller on Day 2 (interfering day) than on Day 1 (*p* = 0.02), indicating some rating adaptation. No main effects relevant to group and day were found, indicating a similar imaging processing level between groups and days.

**Figure 4:**
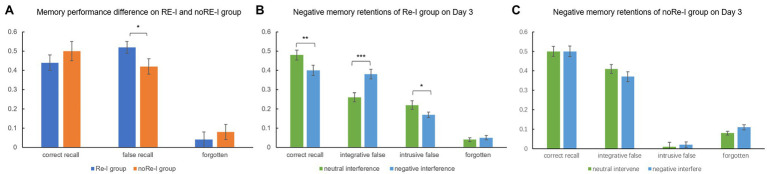
Negative memory performance among two groups and different memory outcomes under neutral and negative interference (Experiment 2). **(A)** The replication of Experiment 1, in which the Re-I group showed higher false memory; **(B)** in the Re-I group, for original negative memories, negative interference materials produce lower correct recall and higher integrative false recall, while neutral interference materials produced higher unabridged invasion (intrusive false recall) to the original memory; **(C)** the noRe-I group did not show the valence related effect for interfering outcomes. The error bar in the figure represents the standard error, * represents *p* < 0.05, ** represents *p* < 0.01, and *** represents *p* < 0.001.

Independent *t*-tests indicated that the false recall rate of the Re-I group (0.52 ± 0.18) was significantly higher than that of the noRe-I group (0.42 ± 0.22), *t*_(74)_ = 1.91, *p* = 0.049, while the correct recall difference between two groups (Re-I group: 0.44 ± 0.19; noRe-I group: 0.5 ± 0.23) did not reach the significant level *t*_(74)_ = −1.11, *p* = 0.271, since the noRe-I group tended to declare more forgotten items (Re-I group: 0.04 ± 0.06; noRe-I group: 0.08 ± 0.07). These results replicated the results observed in Experiment 1, see Figure 4A.

Two (origin valence: neutral/negative) × two (interference valence: neutral/negative) repeated measure ANOVA within the Re-I group revealed a significant interaction between origin valence and interference valence on correct recall, *F*_(1,34)_ = 5.26, *p* = 0.028. A simple effect analysis showed that, for negative memory/origin, the effect of negative interference was significantly better than the effect of neutral interference, resulting in lower correct recall rates (*t*_(34)_ = 3.13, *p* = 0.004).

Interestingly, the condition of valence combination had a distinguishable effect on two types of false recalls (integrative false recall and intrusive false recall). We found a significant main effect of interference valence *F*_(1,34)_ = 16.93, *p* < 0.001, and a significant interaction between origin valence and interference valence on integrative false recall, *F*_(1,34)_ = 4.76, *p* = 0.036. A decomposed simple effect analysis revealed that negative interference exerted significantly richer integrative false to negative memory/origin than that of neutral interference (*p* < 0.001). For intrusive false recall, however, the opposite main effect of interference valence was revealed, *F*_(1,34)_ = 9.2, *p* = 0.005: neutral rather than negative interference invaded into the original memory more, in an indiscriminate manner, see Figure 4B for visualization. As a supplementary analysis, we also performed a repeated measure ANOVA within the noRe-I group. However, it should be noted that, in the noRe-I group, individuals learned a completely new scenario on Day 2 with no targeted interferences. The results showed no significant effects related to valence *F*s < 1.5, *p*_s_ > 0.05, see Figure 4C.

### Discussion

In this experiment, the first finding was that new learning with a negative valence exerted a greater interference effect on negative original memory. This was demonstrated by lower correct recall rates and richer integrative false on the NegO-NegI condition, where the participants attempted to retrieve original negative memories but the memory was reactivated and interfered *via* negative materials on the second day. However, we also found that source confusion intrusion (i.e., intrusive false recall) does not show a valence consistency effect and that common neutral interference is more likely to be confused into the previous memory.

Post-retrieval memory distortion can reflect in different manners. Consistency in the negative valence between the interference material and the original scene can promote integration between the original memory and the novel material, thus leading to the production of more integrative false of the original memory. Meanwhile, the customary and later neutral interference can be easier to express and can more cunningly invade the primitive memory, causing memory updating. The nature of interference materials should be seriously considered.

Interestingly, these valence-related effects were only found in the Re-I group, and consistent with Experiment 1, the Re-I group showed relatively more false reports than the noRe-I group, indicating that memory retrieval plays an important role in the impact of subsequent new learning on original memory modification.

Nevertheless, in addition to integrative reconsolidation, the data can also be applied to other alternative explanations, such as interference theory, interactions between emotions, and false memories. These aspects will be covered in the general discussion.

## General Discussion

In this study, we developed a trial-by-trial interfering reconsolidation paradigm with a self-episodic simulation process to exert better control over memory characteristics while also containing the component of self-processing and context-dependency of episodic memory. We performed two experiments to (1) investigate the possibility of negative declarative memories reconsolidation when self-simulation was served as the learning/interfering manner; and (2) explore how the emotional valence of memory/interference materials affects this reconsolidation process. The results revealed that negative episodic memories can be degenerated and updated in the paradigm. Individuals whose memory was evoked by the old cueing object and who then learned the novel scene combinations generated more false memories than individuals who were not reminded or those whose old memory was reactivated but with no subsequent interference. These results indicated that both the reactivation of original memories and the input of new information are essential components for memory reconsolidation (Experiment 1). However, the degree and the manner of memory distortions that occurred were affected greatly by the nature of the new information being presented after the reactivation. For negative memories, new learning with the same valence (i.e., negative) was found to induce a richer, integrative false recall and resulted in a lower correct recall. Such a finding indicates that in order to optimize negative memory distortions, new information with a certain degree of similarity and integrability to the original memory trace is required. Meanwhile, habitual and neutral interference showed a higher ability to invade into the original memory in a complete form, suggesting that post-retrieval memory integration and intrusion may underline different functional mechanisms (Experiment 2). Also, explanations beyond the reconsolidation framework should be considered to provide a more comprehensive perspective of these phenomena, such as the traditional retroactive interference effect and the influence of emotions on false memories. In any case, the attempt to update negative self-memories using the post-retrieval learning procedure seems promising but also requires further in-depth explorations.

It is undeniable that in the ecological scenario, the formation, storage, and retrieval of episodic memory cannot exclude the influence of self, given *self* is indeed the subject and the main coding/retrieval framework of a personal memory (Conway and Loveday, 2015). The explicit self-referential imagination conducted in the learning task, to some extent, may simulate autobiographical memory processing, which makes the memory in the laboratory more self-ecological. Indeed, memory generated by self-referential processes usually results in better memory performance than other types of coding strategies and exhibits a certain resistance to forgetting or errors (Klein and Kihlstrom, 1986; van den Bos et al., 2010; Klein, 2012). Self-referential negative memories are no exception in this respect (Yang et al., 2013; Mao et al., 2017). In this study, we found that negative episodic memory reinforced *via* the self-simulation process can be updated by new self-referential interfering after the original memory trace is reactivated. These observations may indicate some superiorities of the reconsolidation framework for maladaptive memory updating (Chan and LaPaglia, 2013; Wichert et al., 2013), compared with suppression or directed forgetting strategies for self-memory.

Indeed, unlike the traditional forgetting/suppression paradigm (adding self-reference will reduce forgetting), in the retrieval-interfering processing, self-involvement or self-agency to constructing can instead be the key factor to induce memory change. We found that, in the Re-I group, the self-involvement rating on the post-retrieval phase (Day 2) was positively correlated with the final false recall, suggesting that subjective constructive processing can profoundly affect the characteristics of original memories. This is in line with several studies indicating that self-reference can facilitate false memory just as strengthening the coding, possibly by boosting the gist-based processing (Wang et al., 2019). However, as an extra reminder, this relationship of self-processing and false memory was only observed in the Re-I manipulation, which means that the effect of self-involvement on memory changes should depend on the retrieval procedure. Both primitive memory reactivation and initiative construction are critical links for self-memory updating.

In Experiment 2, we further revealed that certain types of interference materials after reactivation can achieve greater levels of self-memory distortions. Specifically, we found that, for negative memory, interference by negative materials generated an optimal effect, as demonstrated by a greater tendency to make false recall for the original memory. Notably, these false recalls were not dominated by simple intrusions from Day 2 learning, but somehow composed of combination between original memory and subsequent interference information (e.g., a participant learned a gun on the first day and a burning house on the second day; after the old background presentation, he reported “a bomb” in the recall tests for the first-day memory). It might epitomize the integration of memory, since a higher conceptual level (e.g., the war) was activated (Anderson, 1983), with self-referential imagination may provide the conditions for this activation expansion (Wang et al., 2019). Indeed, previous studies have also provided similar evidence that new negative learning (but not neutral or positive one) can lead to greater updating of the negative autobiographical memory. For example, Kredlow and Otto (2015) found that only a negative story exerted a significant interference effect (i.e., lower memory accuracy) on collected trauma-related (negative) memories from the real world compared to neutral or positive materials.

These results indicate that, for an original memory to be modified, the interference information needs to be a “familiar stranger.” That is, the material must be necessarily novel but, at the same time, must also have a feeling of familiarity to be grafted with the original memory formation. The consistent valence between old and new learnings may activate a broader common conceptual network and increase the probability of assimilation of reactivated experience (Anderson, 1983). This process results in the neglect of trivial details of memory retention, so as to get a more coordinated gist (Schacter et al., 2011), which thus seems consistent with the adaption perspective of memory reconsolidation (Bermudez-Rattoni and McGaugh, 2017).

Nevertheless, for the superior interference effects of negative material, alternative explanations should also be noted. One is about the implication between emotion and false memory. In fact, studies have found that high arousal emotion states or learning materials can induce more false memory regardless of their valence features (Corson and Verrier, 2007), basically because the high arousal contents or states narrow the attention scope when encoding, leading to concentrating on gist trace while ignoring peripheral details (Corson and Verrier, 2007; Kaplan et al., 2015; Bookbinder and Brainerd, 2016). Although relevant conclusions are, in general, derived from single-consolidation tasks, similar phenomena may also exist in the process of reconsolidation. For instance, in the study of Wang et al. (2021), the interference of positive materials after memory reactivation led to higher false memory than neutral materials. Combined with the findings that post-retrieval negative material generated more integrative false, it can be assumed that emotional involvement after retrieval may affect original memory quality. Indeed, there is also ample evidence that the intake of stress hormones (with artificial high arousal) or stress manipulation after reactivation can substantially impact original retention, even if the direction of influence (enhance or impair) holds some inconstancy in different memory systems (Zhao et al., 2009; Schwabe and Wolf, 2010; Bos et al., 2014a,b; Wood et al., 2015). However, it should be pointed out that the black-and-white materials used in this study may be not sufficient to generate extremely high arousal. Therefore, the effect of valence consistency might be more dominant. The objective measurement of both valence and arousal ratings of materials would be quite important for future exploration.

The requirement that modification of negative memories requires the involvement of negative interference would appear to pose a challenge to any future intervention therapy. Does such a finding mean that any effective intervention would need to dilute one catastrophe with another? Such speculation has some support. One study, for example, looked at young people who had experienced natural disasters. They found that individuals who had experienced both Hurricane Katrina and Hurricane Gustav (a similar but less severe storm) could remember less details of Hurricane Katrina over time than a group of peers who had experienced only Hurricane Katrina (Weems et al., 2014). Such findings suggest a role for a secondary but weaker negative experience, which, by evoking similar context cues, can reactivate a previous suffering. Controlled new negative events accompanied by less catastrophic consequences could then interfere with initial experiences and thus reduce emotional load. Interestingly, in the study of Wang et al. (2021), positive updating materials impaired negative memory retention but failed to reduce (even strengthen) the emotional significance of negative memories. This may be related to some unpleasant cognitive conflicts caused by excessive prediction errors (Sinclair and Barense, 2019). Therefore, it may be worth trying to introduce consistent rather than countered traces to omitting negative significance in a gradual manner. In fact, in clinical practice, gradual exposure therapy like systematic desensitization has long been considered the strategy of fighting aversive past with aversive current (Wolpe, 1961). Nevertheless, by providing converging points that can be integrated with the original trace, well-designed materials, regardless of simply negative valence, might also achieve such effect to update, only when the memory relevance can be maintained (Lee, 2009).

In the data, neutral rather than negative post-learning scenes seemed to be more likely to win the competition for memory expression and embed themselves in the previous target memory (the intrusive false recalls). The original connection between a specific context (the cueing pictures) and a negative event on the first day was weakened as a result of learning a stronger neutral association link. Based on this mechanism, the impact of post-retrieval processing on the original memory is not sensitive to the homogeneity of new and old information but is more dependent on the depth of interference learning.

Indeed, such interference effects, including the interference from negative materials, may be independent of the reconsolidation process as a consequence of simultaneous retrieval interference (Forcato et al., 2007). In other words, a certain memory may not be updated or modified but damaged in competition with another independent memory trace. In the paradigms considered, the expression of two presumed independent memory traces (i.e., A–B and A–D) can be competing given the common/simultaneous reminder cue (i.e., A). This “retrieval competition” can lead to the observation of retrieval impairment in previous memories, regardless of the updating mechanisms. Indeed, there are a number of evidence that the results generated by reconsolidation do not go beyond the interpretation framework of interference theory (Klingmuller et al., 2017), in which expression competition in memory retrieval is somehow inevitable (Earhard, 1976).

Nevertheless, even we cannot give an exact answer as to whether the memory distortion comes from the reconsolidation-updating of a single memory trace or the result of an expression failure during trace competition, and the experiments substantially proved that post-reminder/retrieval learning can be the strategy to facilitate updating or interfering. The inference effect was mainly restricted in the Re-I group as shown in these data. For example, the positive correlation between self-rating and memory changes was observed in the Re-I group (exp.1), and the noRe-I group did not show valence-related effects in Experiment 2. These results assume the unique role of reactivation/reminder on memory dynamics (Alberini, 2005).

One caution that should be noted is that, in this research, we regard memory distortion as an indicator of memory update, given the adaptive perspective of false memory. Indeed, several types of false memory reflect adaptive cognitive processes that contribute to the efficient functioning of memory (Schacter et al., 2011). The adaption significance of memory is to simulate and predict future events rather than record verbatim details of an entire experience (Suddendorf and Corballis, 2007). In the field of autobiographic memory, highly superior autobiographical memory (HSAM or hyperthymesia), which refers to a syndrome that people are able to remember an abnormally large number of their life experiences with very high accuracy, can be defined as a maladaptive memory phenomenon (Parker et al., 2006; LePort et al., 2012). Representation of the Gestalt principles of “coherence” and “correspondence” on self may inevitably introduce some memory false (Conway et al., 2004).

Still, using reconsolidation to induce false memories seems to be unethical and may cause problems in real life. A reasonable intervention goal should be to impair the feasibility of negative memory reappearance. In the experiments carried out in this study, individuals were encouraged to report any stored impressions in the testing phase, and reports that did not match the standard answers were classified as false recalls, in which a considerable part was consistent with impaired memory trace, combined with some low-confidence ambiguities. However, we did not recollect objective measures of confidence degree for the answer reports. Future research can consider this deficiency, so that the characteristic of memory outcomes (e.g., low-confidence false recall, high-confidence false recall, low-confidence correct recall, high-confidence correct recall) can be meticulously examined.

Besides, other limitations of this study also need to be stated to guide future research. First, even if the research paradigm emphasized the components of explicit self-consciousness (self-referential simulation) on memory processing, the memory materials *per se* are still based on drawing pictures. In order to further improve the ecological validity of memory contents, some real-time event collection techniques can be employed, such as recording real-life senses through a camera as further memory materials (St Jacques and Schacter, 2013) or employing virtual reality scenes (Smith, 2019). Relevantly, we used the term episodic memory to identify the memory form in this study, because the paradigm is assumed with constructive and contextual components and is compared with semantic memory that only includes generic, context-free knowledge (Tulving, 2002; Wheeler and Ploran, 2009). However, in order to better emphasize the nature of episodic memory rather than simply associated pair, the process of encoding and interference can contain the depicting of temporal/spatial relationships between materials, accompanied with self-simulation, to better form an immersive first-perspective episodic event (Wang et al., 2021). Second, in the process of self-simulation, participants were only required to report the degree of self-involvement in imagination, but no more measures on the imaginability were required, which may have a profound impact on the depth of encoding and interfering processing. So, even if the materials have been independently rated during the practical self-simulation task, it would be helpful to include more subjective evaluation indicators like imaginary difficulty and imaginary vividness. Third, sleep can deeply affect reconsolidation processing (Moyano et al., 2019), but this study did not collect data on the sleep state of the participants in the experiment and the time interval between learning/testing and sleeping. Future research can note and record these data to investigate the interesting interaction between reconsolidation and sleep, especially when emotional factors are involved.

Despite all these limitations, the findings provide a glimpse of possibility that negative episodic memory can be updated and integrated *via* reactivation-interfering, in which the valence characteristics of interfering materials can modulate the quality and quantity of memory updating. Besides, even if the original memory can be damaged and modified by the procedure of post-retrieval interference, it may contain different mechanisms on these memory changes. The paradigm could be applied in a number of ways to deepen the understanding of episodic memory reconsolidation. Especially, in the trial-by-trial episodic-simulation manipulations, memory contents could be well controlled but remain as the elements of self-processing. Thus, future cognitive neuroscience approaches (e.g., electroencephalogram and functional magnetic resonance imaging) can be easily applied to explore the underlying mechanism of post-retrieval memory interfering as well as the role of self in this process. Besides, examining individual differences (e.g., how the level of anxiety/depression modulates the degree of self-memory updating) would also be necessary and possible in order to develop effective memory-targeted interventions.

## Data Availability Statement

The raw data supporting the conclusions of this article will be made available by the authors, without undue reservation.

## Ethics Statement

The studies involving human participants were reviewed and approved by the Ethics Committee of the Institute of Psychology, Chinese Academy of Sciences. The patients/participants provided their written informed consent to participate in this study.

## Author Contributions

D-NP was responsible for the design and execution of the experiment, data analysis, and article draft writing. XL was responsible for the experimental design, supervision, and revision of the article. All authors contributed to the article and approved the submitted version.

### Conflict of Interest

The authors declare that the research was conducted in the absence of any commercial or financial relationships that could be construed as a potential conflict of interest.

## Footnotes

^1^https://godotengine.org/

^2^https://godotengine.org/
